# Diagnosis of Skin Burn-Induced Colon Circulatory Disorders Using Optical Coherence Tomography Angiography and Laser Doppler Flowmetry (Experimental Study)

**DOI:** 10.17691/stm2024.16.2.05

**Published:** 2024-04-27

**Authors:** M.G. Ryabkov, P.V. Peretyagin, S.A. Shestakova, S.S. Ptushko, M.S. Koshmanev, Y.L. Bederina, A.L. Potapov, M.A. Sirotkina, N.D. Gladkova, E.B. Kiseleva

**Affiliations:** MD, DSc, Associate Professor, Chief Researcher, Laboratory of Optical Coherence Tomography, Institute of Experimental Oncology and Biomedical Technologies; Privolzhsky Research Medical University, 10/1 Minin and Pozharsky Square, Nizhny Novgorod, 603005, Russia; Junior Researcher, Department of Physico-Chemical Researches, Central Research Laboratory; Privolzhsky Research Medical University, 10/1 Minin and Pozharsky Square, Nizhny Novgorod, 603005, Russia; Student; National Research Lobachevsky State University of Nizhny Novgorod, 23 Prospekt Gagarina, Nizhny Novgorod, 603950, Russia; Student; Privolzhsky Research Medical University, 10/1 Minin and Pozharsky Square, Nizhny Novgorod, 603005, Russia; Medical Resident; Privolzhsky Research Medical University, 10/1 Minin and Pozharsky Square, Nizhny Novgorod, 603005, Russia; Pathologist, Junior Researcher, University Clinic; Privolzhsky Research Medical University, 10/1 Minin and Pozharsky Square, Nizhny Novgorod, 603005, Russia; PhD Student, Laboratory Technician, Laboratory of Optical Coherence Tomography, Institute of Experimental Oncology and Biomedical Technologies; Privolzhsky Research Medical University, 10/1 Minin and Pozharsky Square, Nizhny Novgorod, 603005, Russia; PhD, Director of the Institute of Experimental Oncology and Biomedical Technologies; Privolzhsky Research Medical University, 10/1 Minin and Pozharsky Square, Nizhny Novgorod, 603005, Russia; MD, DSc, Professor, Head of the Laboratory of Optical Coherence Tomography, Institute of Experimental Oncology and Biomedical Technologies; Privolzhsky Research Medical University, 10/1 Minin and Pozharsky Square, Nizhny Novgorod, 603005, Russia; PhD, Senior Researcher, Laboratory of Optical Coherence Tomography, Institute of Experimental Oncology and Biomedical Technologies; Privolzhsky Research Medical University, 10/1 Minin and Pozharsky Square, Nizhny Novgorod, 603005, Russia

**Keywords:** skin burn, colon, microcirculation, non-occlusive ischemia, optic coherence tomography angiography, laser Doppler flowmetry

## Abstract

The condition of gastrointestinal tract determines in many respects the regenerative capacity and the risk of complications in patients with extensive skin burns. However, the mechanism of developing vascular dysfunction in the colon in the burned individuals has so far been poorly studied.

**The aim of the investigation** is to study intramural circulatory disorders of the colon using optical coherence tomography angiography (OCTA) and laser Doppler flowmetry (LDF) in different time periods after modeling a thermal burn.

**Materials and Methods:**

A deep thermal skin burn was induced on the area covering 10% of the body surface of Wistar rats (n=15). The blood flow of the colon wall was continuously monitored for 15 min before and 45 min after the burn using OCTA and LDF. The colon wall was again studied on days 7 and 14 using the same OCTA and LDF techniques. At each time point (45 min, day 7 and 14), 5 animals were withdrawn from the experiment, the colon wall was taken for histological study. The colon wall samples from three control rats without thermal skin burns were also histologically investigated.

**Results:**

During 45 min after the induction of the thermal burn, the *in vivo* OCTA and LDF techniques registered changes in intramural blood flow in the form of dropping of some arterioles and capillaries out of the general blood flow with concurrent activation of vascular shunts as a compensatory mechanism. Histologically, a marked edema of the submucosa, erythrocyte aggregation, and stasis in the capillary network were observed in this period. According to the OCTA and LDF data, the microcirculatory disorders in the colon were partially resolved by day 7, and by day 14 the analyzed indicators returned to the initial level. The data of the histological evaluation have shown that on day 7 after the burn induction, submucosal edema was absent, however, the signs of microcirculatory disorder and inflammatory changes remained. On day 14, the pathological changes in the tissues were not observed.

**Conclusion:**

The OCTA and LDF methods allowed us to establish experimentally that during the first 45 min thermal burn causes considerable disturbances of the blood flow in the colon wall, which normalizes only by day 14 if no therapy is administered. The obtained data on the mechanism of circulatory disorder development in the colon may become a basis for choosing therapy directed to prevention of intestine dysfunction in people with burns.

## Introduction

Acute skin burn injury causes damage to a number of organs and systems anatomically distant from the covering tissues. One of such target systems is a gastrointestinal tract (GIT), the damage of which worsens the prognosis of patients with thermal burns [1]. The time and quality of burn wound healing depend on the functional activity of the GIT at all stages of the burn disease [2]. Besides, among patients with extensive thermal burns the occurrence of GIT dysfunction reaches 40–45% [3]. In the proximal parts of the GIT, skin burn is accompanied by stimulation of gastric secretion, ulceration of the stomach/duodenum mucosa, inhibition of peristalsis and intestinal absorption [4]. It is known from clinical observations that dysfunction of the distal GIT part in patients with burns manifests itself by pseudo-obstruction of the colon, sepsis and abdominal compartment syndrome, increase of colonic mucosa permeability, and bacterial translocation [1]. Late diagnosis, absence of objective data of the colon wall condition result in immediate surgical interventions [5]. More than 80% of the dead patients with extensive skin burns in the clinical picture were noted to have GIT dysfunction: diarrhea, constipation or nausea/vomiting, acute gastroduodenal ulcers, and bleeding [6].

The colon is an important anatomical part of the digestive tract determining the risk of developing severe complications in burned patients. The course of colon pseudo-obstruction (dynamic intestinal obstruction) in burned patients is aggressive and prognostically much more unfavorable than in patients of other nosological groups [5]. The functional condition of the colon, presence of dynamic obstruction or diarrhea strongly correlate with the risk of sepsis development in the burned people [7].

Despite an important role of preserving colon functions during the burn disease, little is known about the nature of structural and intramural circulatory disorders of this organ in the burned individuals. The available data on the role of mesenteric ischemia in the pathogenesis of the burn disease concern mainly a small intestine: the frequency of non-occlusive acute mesenteric ischemia (NOMI) in the basin of the superior mesenteric artery in the burned patients is 1–2%, and the risk of this complication grows with the increase of the thermal injury area. The lethality rate in the burned patients with acute mesenteric ischemia (90 days) is two times higher than that of the level in patients without it at equal values of the burn injury area and depth [3]. Refractory hypovolemia and reduced cardiac output, which are more typical for patients in shock condition, burn or septic, are known to trigger NOMI of the small intestine [8]. However, information on the circulatory condition in the colon wall in patients with skin burns is much scarcer. Specific details of pathogenesis of intestine ischemia with area-limited skin burns also remain insufficiently explored. New knowledge in this field is important for the choice of time and target therapy configuration, prevention of vascular disturbances in the intestine in a large group of burned patients.

One of the technical problems hampering the study of the nature of pathological changes in the colon wall in burned people is absence of diagnostic technologies for a long-term monitoring of blood circulation and intestine wall structure. Adaptation of optical coherence tomography angiography (OCTA) to the application in the abdominal cavity on the contracting gut *in vivo* [9] allows for making a significant progress in solving this problem. Previously, OCTA has already been used for visualization of microvascular bed of various parts of the digestive tract: esophagus [10, 11], stomach [12, 13], small intestine [14, 15], for investigation of experimentally modeled pathology, and in practical diagnostic activity, however, this method has not been employed to diagnose the colon condition on the experimental models of skin burn injury.

Laser Doppler flowmetry (LDF) is one of a few methods of diagnosing blood circulation in the intestine wall included into the Russian clinical recommendations [16]. The diagnostic capacity of the LDF technology allows for real time verification of blood flow parameters in the tissue *in vivo* and determining the main pathogenetic mechanisms of circulatory disorders [17], for example, in inflammatory intestinal diseases [18] and intestinal ischemia [19, 20].

A complex application of OCTA and LDF for blood flow monitoring of the colon wall makes it possible to assess functional indicators of microcirculation and the amount and morphologic parameters of functioning intramural vessels at any given moment, to obtain new knowledge about pathogenic mechanisms of circulatory disorders in the intestine in thermal skin burns. Taking into account variability of vascular network architecture in the intestine wall and a “delicate” character of possible changes in the visceral circulation in thermal skin injury, it is advisable to acquire data from OCTA and LDF concurrently from the same zone of the intestine wall. Previously it has not been possible due to a large number of motion artifacts and difficulty of OCTA probe fixation. However, the device for vacuum fixation of the OCTA probe to the tissue surface, designed by us, provided the acquisition of a series of high-quality OCTA images from one examined point in dynamics [9]. Thus, presently, it became feasible technically and methodologically to solve clinically and fundamentally significant task of exploring the reaction of microcirculatory colon bed for the presence of an extensive thermal burn in patients.

**The aim of the research** is to study intramural circulatory disorders of the colon using optical coherence tomography angiography and laser Doppler flowmetry in different time periods after modeling a thermal burn.

## Materials and Methods

The experimental study was performed at the Institute of Experimental Oncology and Biomedical Technologies of Privolzhsky Research Medical University (Russia). The study was approved by the Ethical Committee of Privolzhsky Research Medical University (protocol No.12 of August 05, 2022). Male Wistar rats weighing 150‒215 g were used (n=18, of them 15 animals represented a group of rats with a thermal burn and 3 were in the control group). Animal housing in the vivarium and the research work were carried out in accordance with Guide for the Care and Use of Laboratory Animals and European Convention for the Protection of Vertebrata used for Experimental and Other Specific Purposes (Strasburg, 2006). Surgical interventions were conducted under general anesthesia with a mixture of the following solutions: 0.2 ml of 3.5% tiletamine hydrochloride + zolazepam hydrochloride, and 0.1 ml of 2% xylazine hydrochloride introduced intraperitoneally.

**Figure 1.**
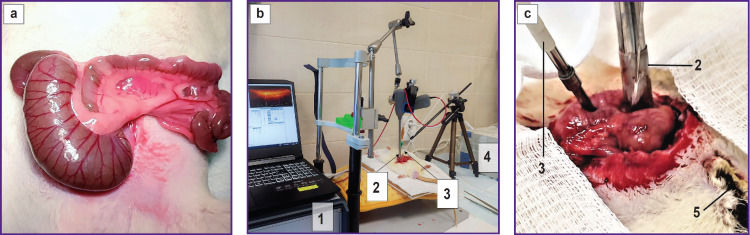
Experimental design: (a) day 1: the examined segment of the colon was brought out through the laparotomic wound; (b) day 1: OCTA and LDF data acquisition prior to burn wound induction; (c) day 7: relaparotomy, OCTA and LDF data acquisition. *1* — multimodal OCT system; *2* — OCT probe is in contact with the colon surface; *3* — LASMA ST sensor is in contact with the colon surface; *4* — LASMA ST device for obtaining LDF data; *5* — a segment of thermal skin burn on the left lateral surface of the abdominal wall

Under aseptic conditions of the experimental operating-room and after anesthetizing, mid-median laparotomy was performed. A segment of the colon was brought out through the wound (Figure 1 (a)), the OCTA and the LDF probes were placed on the colon surface on the side of the serosa at a distance of 3–4 mm from each other (Figure 1 (b)), blood flow monitoring was carried out for 15 min. In this way, a continuous series of OCTA and LDF data were captured on the condition of the microcirculatory bed at the neighboring points of the selected colon zone before the induction of the burn injury. Next, a deep thermal skin burn was modeled on the left half of the abdominal wall covering 10% of the animal body surface. The burn area in the experimental model was selected to reproduce a clinical picture in a maximally wide group of sufferers with thermal burns and at the same time to minimize the probability of generalized hemodynamic disorders typical for the burn shock.

While inducing a thermal burn and during subsequent 45 min, OCTA and LDF monitoring of the colon blood flow was continued. Then, the examined tissue samples were taken from 5 animals for histological examination, in 10 animals the laparotomic wound was sutured. The animals were left to live in the standard vivarium conditions; no therapy of the burn wound was administered. On day 7 after the primary intervention, half of the animals (5 of 10), and on day 14, the rest of 5 animals underwent relaparotomy under anesthesia and again OCTA and LDF data on colon blood circulation in the previously examined segment were being received for 45 min (Figure 1 (c)). The region of interest was normally located on the anterior surface of the proximal part of the colon 1.5 cm from the ileocecal junction, therefore, was readily identified at the next stage of the study. After the diagnostic procedure, a portion of the colon was taken for histological study. Animals were withdrawn from the experiment due to muscle relaxant overdose. Colon wall samples were harvested from three control rats without skin burn wounds for histological examination.

The OCTA and LDF methods were used to monitor the microvascular bed of the colon wall. The OCTA technique, applied to monitor the condition of intramural vessels of the colon by their visualization before and at different time points after the induction of the burn wound, was implemented with the help of the spectral multimodal OCT system (Federal Research Center Institute of Applied Physics of the Russian Academy of Sciences, Russia). The system has the following technical characteristics: 1310 nm wavelength of the probe radiation; 10 μm lateral resolution; 15 μm depth resolution; 1.7 mm scanning depth; 20,000 A-scans/s scan rate. A 10 mm outer diameter probe was placed in contact with a lateral surface of the colon and held fixed for 26 s to record one OCT image. A continuous scanning was performed along the fast axis (512 spectral measurements, 256 spectral A-scans) and along the slow axis (1024 B-scans), the data were combined into a volumetric 3D image (4×4×2 mm). 2D *en-face* images of the vascular networks were built in the process of OCT data acquisition by automatic high-frequency OCT signal filtration: moving scatterers (erythrocytes motion in the blood vessels) were well distinguished against the background of the stationary scatterers (tissue surrounding blood vessels).

The OCTA image analysis included their visual and quantitative assessment. For quantitative analysis calculation of the total length of all vessels (*L*, μm) and also a portion of vessels with a diameter of ≤15 μm (*L*1), 16 — 60 μm (*L*2), 61 — 100 μm (*L*3), and over 100 μm (*L*4) of the total length of the vascular bed was performed. Computations were done using original program written in the Anaconda 4.3.1 (Python v. 3.6) mathematic medium.

Laser Doppler flowmetry was carried out using a laser diagnostic system LASMA ST (NPP LASMA, Russia). A sensor was placed on the serosa surface of the colon at a distance of 3–4 mm from the OCT probe. The analysis of the LDF (a scanning volume is 1.5 mm^3^) data included computation of MI, microcirculation indicator (perfusion), i.e. a mean blood volume in the specified time interval measured in arbitrary perfusion units (p.u.); MSHUNT, shunt component of perfusion, i.e. the ratio of neurogenic and myogenic oscillation amplitudes of the vascular wall measured in the relative units (r.u.).

To perform a histological examination at each stage (45 min, day 7, day 14), 5 animals were withdrawn from the experiment; parts of the colon wall, examined by OCTA and LDF and marked with a histological stain, were taken for the histology. The tissue samples were fixed in 10% formalin. After fixation, the samples underwent standard histological processing in the Excelsior ES tissue processor (Thermo Scientific, USA). Next, the tissues were oriented according to the regions of OCTA and LDF scanning, and embedded into the paraffin blocks using the HistoStar tissue embedding system (Thermo Scientific, USA). Series sections of 3–4 μm thick were cut using the Microm HM 325 microtome (Thermo Scientific, USA). The sections were stained with hematoxylin and eosin in the Gemini AS slide stainer system (Thermo Scientific, USA). For morphometric processing and creation of a photo archive of the material obtained we used the Leica 2500 microscope (Leica Microsystems, Germany) and a scanning complex 3DHISTECH PANNORAMIC Midi (Carl Zeiss, Germany) in the light field mode with 50–400 magnification and application of a computerassisted image analysis system.

### Statistical data processing

The data were statistically processed using the IBM SPSS Statistics v. 20 program. Statistical significance of differences between the groups by the qualitative features was evaluated using Mann–Whitney and Kruskall–Wallis tests. The sample parameters were designated in the following way: Me — median, Q1 — upper quartile, Q3 — lower quartile, n — volume of the analyzed subgroup, p — value of statistically significant differences, p_adjusted_ — corrected value of statistically significant differences with Bonferroni correction in multiple comparisons. The critical value of the significance level was taken as 5% (p≤0.05).

## Results

### Results of OCTA monitoring of intramural colon microcirculation in thermal burns

**Figure 2.**
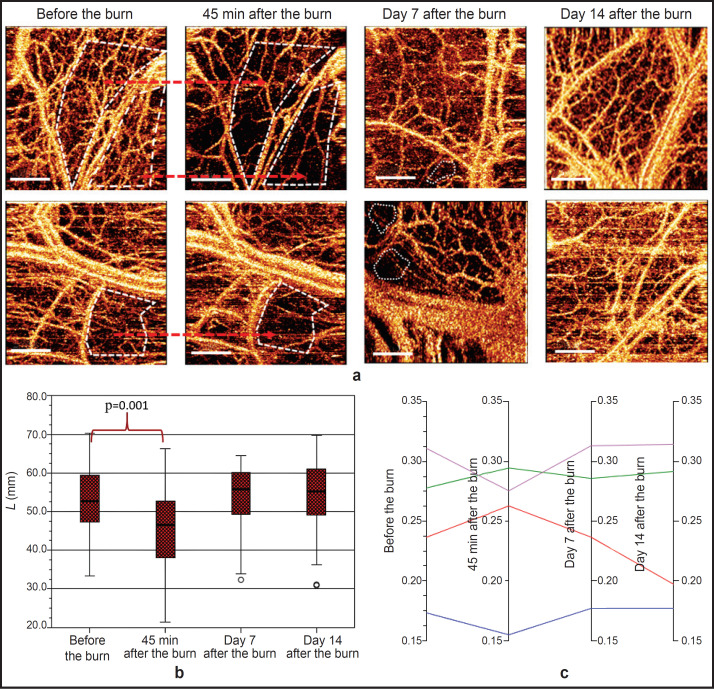
OCTA data demonstrating microcirculatory changes in the colon in different time periods after thermal skin burn modeling: (a) dynamics of vascular network changes in OCTA images in different time periods after thermal burn induction as compared to the blood vessel imaging before the burn. In 45 min after burn induction, vessel-free areas appear in OCTA images (marked by a white dashed line), on days 7 and 14, the angiographic picture is restored; *red arrows* — the regions of OCTA images with specific changes in the vascular bed before and after the burn; bar 1 — mm; (b) dynamics of median values of the total blood vessel length before the burn, 45 min after it, on days 7 and 14 after the induction of the thermal skin burn; (c) dynamics of the proportion of blood vessels of a certain diameter in the overall functioning vascular network: blue line — *L*1 (d≤15 μm); violet — *L*2 (d=16–60 μm); green — *L*3 (d=60–100 μm); red — *L*4 (d>100 μm)

Specific OCTA images were observed for each stage of the experiment (Figure 2 (a)). A dense network of blood vessels of various diameters uniformly distributed across the entire image was visualized before the thermal burn. Immediately after the induction of the thermal injury, pronounced changes began in the colon microvascular bed. Disappearance of a portion of small-caliber blood vessels (less than 60 μm in diameter, i.e. capillaries, arterioles, and venules) and appearance of areas with scarce blood vessels or vessel-free areas with preserved imaging of the large paired vessels (arteries and veins) was a typical pattern of intramural vascular network response to the burn. The quantitative analysis of the OCTA images has shown that the median of the total vessel length before the burn was 52.77 [47.19; 59.62] mm; this indicator decreased to 46.59 [37.27; 53.12] mm (p_adjusted_=0.001) during 45 min after the burn (Figure 2 (b)).

On day 7 after the burn, the visual analysis of OCTA images of the colon wall has shown residual circulatory disorders: vessel-depleted patterns were present but their area reduced considerably. The total length of the blood vessels in the examined regions of the colon was equal to 55.73 [48.08; 60.46] mm, which did not differ statistically significantly from the initial value (p_adjusted_=1.000).

By day 14 after the burn, the total length of the blood vessels was 55.32 [47.94; 62.82] μm and also did not differ from the initial value (p_adjusted_=1.000), and the vascular pattern in all animals was visually similar to that before the induction of the burn injury.

The quantitative analysis of changes in the length of different diameters vessels has shown that the greatest contribution to the reduction of the vascular bed length was made by the vessels with the diameters below 15 μm (*L*1) and 16–60 μm (*L*2): their shares decreased during the first 45 min following the burn from 0.17 [0.15; 0.19] to 0.15 [0.14; 0.18] (p_adjusted_=0.018) and from 0.31 [0.28; 0.34] to 0.27 [0.26; 0.30] (p_adjusted_=0.001), respectively (Figure 2 (c)). At the next time points, on days 7 and 14, these indicators returned to the initial values.

**Figure 3.**
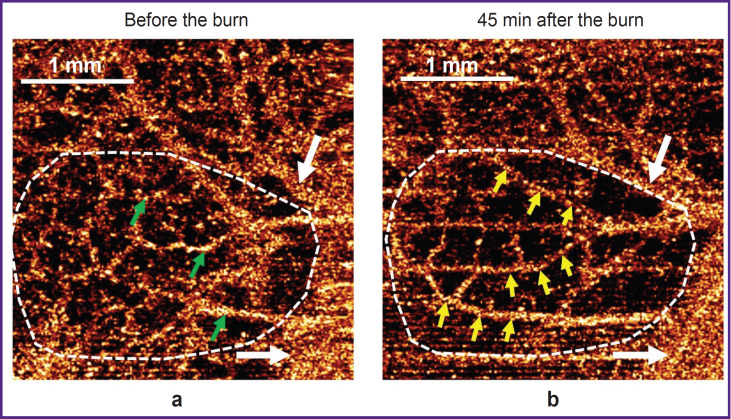
Example of disappearance of small-caliber blood vessels and simultaneous activation of shunt vessels identified in OCTA images of the colon wall 45 min after thermal burn induction (the region of comparison is designated by a white dashed line): (a) before the burn; (b) after the burn induction; *white arrows* — large vessels identical in both images are visualized similarly before and after the burn; *green arrows* — inactive shunt vessels; *yellow arrows* — active shunt vessels

It should be noted that during 45 min after thermal burn induction, other 40–80 μm bridge anastomotic vessels, which connected medium-sized arteries with each other, became more evident in the OCTA images simultaneously with disappearance of the small-diameter vessels in these zones. In Figure 3, the OCTA images demonstrate activation of the shunt vessels: after the burn their diameter enlarged in presence of depleted vascular pattern (*yellow arrows*).

Thus, OCTA monitoring of the colon wall has shown that thermal skin burn was followed by acute disorders of intramural circulation, which were manifested by formation of vessel-free or vessel-depleted areas in OCTA images and signs of vascular shunts activation. The decrease of the total vessels length and formation of the vessel-free areas in the colon wall were due to switching-off the vessels with a diameter less than 60 μm from the blood flow. Their portion in the total length of the perfused vascular bed reduced by 13% (p=0.001).

### Results of LDF monitoring of intramural colon microcirculation in thermal burns

**Figure 4.**
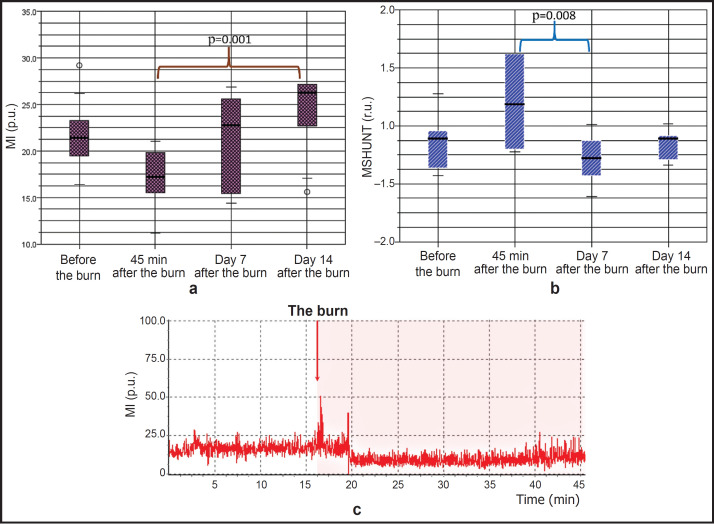
Laser Doppler flowmetry data demonstrating the changes in colon perfusion in different time points after thermal burn modeling: (a) dynamics of MI median values; (b) dynamics of MSHUNT median values; (c) a typical example of LDF-gram changes at the time of inducing a thermal skin burn

The integral LDF parameter, MI, statistically significantly changed throughout the four time points (Kruskal–Wallis test, p=0.001). Immediately after laparotomy prior to thermal burn induction, the MI value was 21.4 [19.3; 23.3] p.u. in the intact wall of the colon, which reduced to 17.3 [15.1; 19.9] p.u. after burn induction, being equal to about 80% of the initial level during the first 45 min (Figure 4 (a)). By day 7 of observation, MI stabilized and made up 22.8 [15.3; 25.7] p.u.; by day 14 it exceeded the initial level and was equal to 26.3 [22.6; 27.2] p.u. Multiple group comparisons of MI values before the burn and 45 min after it showed p_adjusted_ equal to 0.096; before and on day 7 after the burn it was 1.000; before the burn and on day 14 after the burn — 0.102; 45 min after the burn and on day 14 after it — 0.001.

The MI changes after the burn occurred concurrently with dynamic and multidirectional alterations of the shunt component of perfusion (MSHUNT). In the intact colon, MSHUNT was 0.90 [0.57; 1.03] r.u., and during the first 45 min after the burn it grew up to 1.19 [0.80; 1.62] r.u. (Kruskal–Wallis test, p=0.012) (Figure 4 (b)).

These changes were registered on the LDF-gram and remained throughout 45 min at the first stage of the experiment (Figure 4 (c)). By day 7 and 14, MSHUNT stabilized and did not differ statistically significantly from the initial level before the burn (see the table). Multiple intergroup comparisons of MSHUNT parameter have shown p_adjusted_ equal to 0.160 before the burn and 45 min after it; before the burn and on day 7 after the burn it amounted to 1.000; before the burn and on day 14 after it — 1.000; 45 min after the burn and on day 7 — 0.008.

**Table:** Dynamics of the examined microcirculation parameters in the colon wall according to the laser Doppler flowmetry data, Me [Q1; Q3]

Parameters	Before the burn	After the burn		
		45 min	Day 7	Day 14
Microcirculation indicator	21.4 [19.3; 23.3]	17.3 [15.1; 19.9]	22.8 [15.3; 25.7]	26.3 [22.6; 27.2]
Shunt component of perfusion	0.899 [0.569; 1.026]	1.185 [0.803; 1.622]	0.723 [0.532; 0.875]	0.893 [0.698; 0.939]

### Morphological changes in the colon tissue in different terms after thermal burn modeling

**Figure 5.**
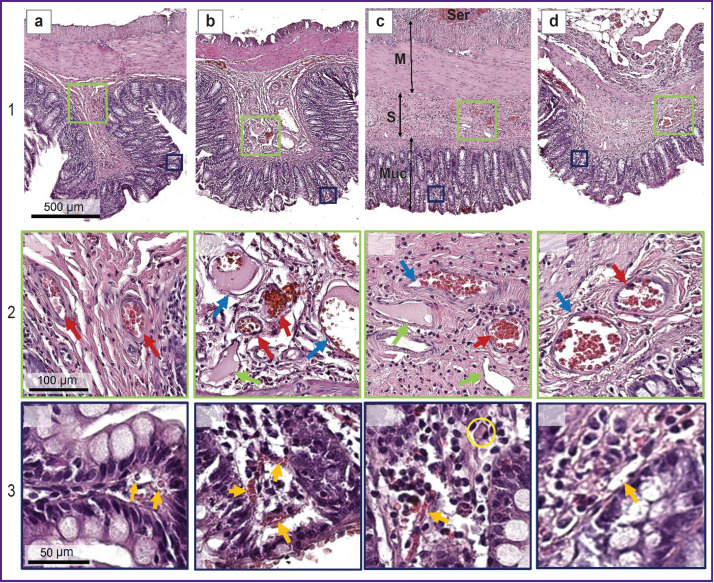
Histological images of the colon wall before burn induction (a), 45 min after (b), on day 7 (c), and on day 14 (d) after the induction of thermal injury: *1* — overview images, the colon wall consists of four layers: a thin serosa (Ser), double-layered muscularis externa (M), submucosa (S), and mucosa (Muc); the colon folds are formed by mucosa and submucosa (a1), (b1); *2* — magnified images of the submucosa with vessels; *red arrows* — arterioles, *blue arrows* — venules, *green arrows* — lymphatic vessels; *3* — magnified images of the mucosa and lamina propria; *orange arrows* — capillaries, a hyaline thrombus in the capillary lumen is designated with a yellow circle

Histological examination of the colon wall before inducing the burn has demonstrated normal layered structure without any signs of inflammation or blood flow disorder. The blood vessels in the submucosa were presented by arterioles and venules (Figure 5 (a1)), arterioles were branching into capillaries at the crypt bottom forming a network in the lamina propria of the mucous membrane (Figure 5 (a2)).

45 min after the burn induction, a marked edema of the submucosa with moderate inflammatory infiltration of lymphocytes and neutrophils was observed (Figure 5 (b1)). Arterioles were plethoric, the venule lumen was widened, with mural hyaline-like deposits, in the lymphatic vessel lumens the lymph was rich with proteins. In the lamina propria, a lot of capillaries with erythrocyte aggregation and stasis were observed (Figure 5 (b2)).

On day 7 after the burn induction, edema of the submucosa was absent, infiltration by neutrophils and lymphocytes remained (Figure 5 (c2)). Arterioles and venules had normal appearance. Single capillaries with erythrocyte aggregation and hyaline thrombi were present in the lamina propria (Figure 5 (c3)).

On day 14 after the burn induction, the structure of the colon wall and vascular bed returned to norm. There were no signs of hyperemia and stasis (Figure 5 (d2), (d3)).

## Discussion

The previous study [3] have shown that blood flow disorders in the mesenteric region play an essential role in the pathogenesis of a burn disease. The prognosis in patients of this nosological group significantly worsens together with the development of acute mesenteric ischemia, and the lethality among them reaching 100%. In observations from combustiologic practice, patients with both non-occlusive (as a rule) and occlusive forms of acute mesenteric ischemia were described. The mechanism of NOMI development with a critical burn area is being actively studied. The NOMI risk is known to grow with the burn area increase [21]. At the early stages of the burn disease, this correlation is likely to be due to the severity of the burn shock, which becomes a trigger of the blood flow redistribution and depletion of the visceral bed. The NOMI risk is especially high among patients with thermal burns complicated by polyorganic insufficiency [22]. Identification of high-risk patients in this population is of critical importance for timely intervention to reduce the risk of lethal outcome. One more clinical situation connected with a high risk of NOMI development in burned people is sepsis and septic shock. The hemodynamics, specific for these states, is a pathogenetic link of circulatory disturbances in the intestine.

The results of our present study confirm the data presented in the introduction section and at the same time essentially deepen and clarify them. It has been established that even a burn of a relatively small area (experimental model with a thermal injury covering 10% of the body surface area), which most commonly does not cause a shock condition, becomes a trigger of acute circulatory disorder in the colon. The OCTA and LDF data have shown that blood flow disorders in the colon wall began to develop immediately after burn induction: changes in the LDF picture occurred during several seconds, the OCTA image changed within several minutes, the histological picture altered within several days. The possibility of such response of the local microcirculatory bed segments not depending directly on the general severity of the burn trauma, has been described in the work [23], however, we showed its manifestations while investigating the digestive tract.

The mechanism of circulatory disorders, defined by us in the colon wall, was probably caused by the reaction of the vascular network to the complex of endocrine and neurogenic disturbances emerging within a few tens of seconds after a thermal burn. The growth of MSHUNT values, established during LDF study, indicates of an increase in contribution of neurogenic blood flow modulation and amplitude of neurogenic oscillations of the vascular wall: the higher the amplitude of neurogenic oscillations and lower the amplitude of myogenic oscillations, the higher MSHUNT values is. The growth of MSHUNT values reflects the reduction of blood volume flowing into the nutritive (interchanging) link of the microcirculatory network of the examined tissue together with dilatation of precapillary sphincters [24]. The data obtained allow us to suppose with a high degree of certainty that the main mechanism of circulatory disorder in the colon wall after thermal skin burn is shunting in the colon wall bypassing the nutritive link of the microcirculatory bed. Previously, LDF has already been employed for diagnosing acute mesenteric ischemia and the informativity of this technique has been validated [25]. However, complex OCTA and LDF data about the manifestations and mechanisms of microcirculatory disorders in the colon in thermal burn were obtained for the first time. OCTA, used by us for verification and imaging intramural colon vessels, made it possible to determine an important mechanism of NOMI development: dropping of microvessels with a diameter less than 60 μm out of the blood flow due to the activation of the vascular shunts. This conclusion is anatomically and physiologically grounded. Blood vessels of the colon are known to run from mesenterium to the submucosa, where they branch in mucosa into a capillary network, muscularis externa and form arteriovenous shunts [26]. Shunting of the arterial blood bypassing the nutritive bed serves as an adaptive reaction, however, as we have shown in our study, it may be followed by pathological consequences: reduction of the perfused microvessel length, activation of arteriovenous shunts, and other acute circulatory disorders in the colon wall, which resolve only on days 7–14 after the induction of the thermal burn if no therapy is administered.

## Conclusion

The OCTA and LDF methods allowed establishing experimentally that already during the first 45 min a thermal burn causes considerable disturbances of blood flow in the colon wall, which normalizes only by day 14 if not treated. The obtained data on the mechanism of circulatory disorder development in the colon may become a basis for choosing therapy directed to the prevention of intestinal dysfunction in people with burns.

## References

[1] Ng J.W. Cairns S.A. O’Boyle C.P. Management of the lower gastrointestinal system in burn: a comprehensive review. Burns 2016; 42(4):728–737, 10.1016/j.burns.2015.08.007 26774605

[2] Yu Y. Zhang J. Wang J. Wang J. Chai J. Effect of blended protein nutritional support on reducing burn-induced inflammation and organ injury. Nutr Res Pract 2022; 16(5):589–603, 10.4162/nrp.2022.16.5.589 36238375 PMC9523203

[3] Soussi S. Taccori M. De Tymowski C. Depret F. Chaussard M. Fratani A. Jully M. Cupaciu A. Ferry A. Benyamina M. Serror K. Boccara D. Chaouat M. Mimoun M. Cattan P. Zagdanski A.M. Anstey J. Mebazaa A. Legrand M. PRONOBURN group. Risk factors for acute mesenteric ischemia in critically Ill burns patients — a matched casecontrol study. Shock 2019; 51(2):153–160, 10.1097/shk.0000000000001140 29561390

[4] Vagner D.O. Krylov K.M. Verbitsky V.G. Shlyk I.V. Prevention of gastrointestinal bleeding in patients with advanced burns. Khirurgiya. Zhurnal im. N.I. Pirogova 2018; 3: 42, 10.17116/hirurgia2018342-48 29560958

[5] John A.A. Anand R. Frost J. Griswold J.A. Acute colonic pseudo-obstruction: a critical complication in burn patients. Burns Open 2022; 6(1):37–41, 10.1016/j.burnso.2021.11.003

[6] He Q.L. Gao S.W. Qin Y. Huang R.C. Chen C.Y. Zhou F. Lin H.C. Huang W.Q. Gastrointestinal dysfunction is associated with mortality in severe burn patients: a 10-year retrospective observational study from South China. Mil Med Res 2022; 9(1): 49, 10.1186/s40779-022-00403-1 36064456 PMC9442990

[7] Dvorak J.E. Ladhani H.A. Claridge J.A. Review of sepsis in burn patients in 2020. Surg Infect (Larchmt) 2021; 22(1):37–43, 10.1089/sur.2020.367 33095105

[8] Yu B. Ko R.E. Yoo K. Gil E. Choi K.J. Park C.M. Non-occlusive mesenteric ischemia in critically ill patients. PLoS One 2022; 17(12): e0279196, 10.1371/journal.pone.0279196 36534676 PMC9762570

[9] Ryabkov M. Sizov M. Bederina E. Zarubenko P. Peretyagin P. Moiseev A. Vorobiev A. Gladkova N. Zaitsev V. Kiseleva E. Optical coherence tomography angiography of the intestine: how to prevent motion artifacts in open and laparoscopic surgery? Life (Basel) 2023; 13(3): 705, 10.3390/life13030705 36983861 PMC10055682

[10] Liang K. Ahsen O.O. Murphy A. Zhang J. Nguyen T.H. Potsaid B. Figueiredo M. Huang Q. Mashimo H. Fujimoto J.G. Tethered capsule en face optical coherence tomography for imaging Barrett’s oesophagus in unsedated patients. BMJ Open Gastroenterol 2020; 7(1): e000444, 10.1136/bmjgast-2020-000444 PMC7473663 32883714

[11] Chu K.K. Zhao Y. Jelly E.T. Steelman Z.A. Crose M. Cox B. Ofori-Marfoh Y. Moussa L. Cirri H. Watts A. Shaheen N. Wax A. Esophageal OCT imaging using a paddle probe externally attached to endoscope. Dig Dis Sci 2022; 67(10):4805–4812, 10.1007/s10620-021-07372-w 35084606 PMC10015416

[12] Zuccaro G. Gladkova N. Vargo J. Feldchtein F. Zagaynova E. Conwell D. Falk G. Goldblum J. Dumot J. Ponsky J. Gelikonov G. Davros B. Donchenko E. Richter J. Optical coherence tomography of the esophagus and proximal stomach in health and disease. Am J Gastroenterol 2001; 96(9):2633–2639, 10.1111/j.1572-0241.2001.04119.x 11569687

[13] Jansen S.M. de Bruin D.M. van Berge Henegouwen M.I. Strackee S.D. Veelo D.P. van Leeuwen T.G. Gisbertz S.S. Optical techniques for perfusion monitoring of the gastric tube after esophagectomy: a review of technologies and thresholds. Dis Esophagus 2018; 31(6): dox161; 10.1093/dote/dox161 29701760

[14] Kiseleva E. Ryabkov M. Baleev M. Bederina E. Shilyagin P. Moiseev A. Beschastnov V. Romanov I. Gelikonov G. Gladkova N. Prospects of intraoperative multimodal OCT application in patients with acute mesenteric ischemia. Diagnostics (Basel) 2021; 11(4): 705, 10.3390/diagnostics11040705 33920827 PMC8071199

[15] Tian Y. Zhang M. Man H. Wu C. Wang Y. Kong L. Liu J. Study of ischemic progression in different intestinal tissue layers during acute intestinal ischemia using swept-source optical coherence tomography angiography. J Biophotonics 2024; e202300382, 10.1002/jbio.202300382 38247043

[16] Russian Society of Surgeons. Ostraya neopukholevaya kishechnaya neprokhodimost’ (klinicheskie rekomendatsii) [Acute non-tumor intestinal obstruction (clinical guidelines)]. Moscow; 2021.

[17] Berge S.T. Safi N. Medhus A.W. Ånonsen K. Sundhagen J.O. Hisdal J. Kazmi S.S.H. Gastroscopy assisted laser Doppler flowmetry and visible light spectroscopy in patients with chronic mesenteric ischemia. Scand J Clin Lab Invest 2019; 79(7):541–549, 10.1080/00365513.2019.1672084 31560225

[18] Sheng L. Hu F. Yu H. Tao X. Jia R. Gu Y. Chen L. Kong H. Miao C. Fei W. Yang Y. Jia J. Zhu X. He X. Hu L. Ma J. Liu W.T. Yang M. Paeoniflorin inhibits ASK1-TF axis by up-regulating SOCS3 to alleviate radiation enteritis. Front Pharmacol 2022; 13: 743708, 10.3389/fphar.2022.743708 35359871 PMC8964139

[19] Yu Q. Yang X. Zhang C. Zhang X. Wang C. Chen L. Liu X. Gu Y. He X. Hu L. Liu W.T. Li Y. AMPK activation by ozone therapy inhibits tissue factor-triggered intestinal ischemia and ameliorates chemotherapeutic enteritis. FASEB J 2020; 34(9):13005–13021, 10.1096/fj.201902717rr 32776374

[20] Tuncer F.B. Durmus Kocaaslan F.N. Yildirim A. Sacak B. Arabaci Tamer S. Sahin H. Cinel L. Celebiler O. Ischemic preconditioning and iloprost reduces ischemiareperfusion injury in jejunal flaps: an animal model. Plast Reconstr Surg 2019; 144(1):124–133, 10.1097/prs.0000000000005708 31246814

[21] Muschitz G.K. Fochtmann A. Keck M. Ihra G.C. Mittlböck M. Lang S. Schindl M. Rath T. Non-occlusive mesenteric ischaemia: the prevalent cause of gastrointestinal infarction in patients with severe burn injuries. Injury 2015; 46(1):124–130, 10.1016/j.injury.2014.08.035 25239541

[22] Kowal-Vern A. McGill V. Gamelli R.L. Ischemic necrotic bowel disease in thermal injury. Arch Surg 1997; 132(4):440–443, 10.1001/archsurg.1997.01430280114020 9108768

[23] Hernekamp J.F. Neubrech F. Cordts T. Schmidt V.J. Kneser U. Kremer T. Influences of macrohemodynamic conditions on systemic microhemodynamic changes in burns. Ann Plast Surg 2016; 77(5):523–528, 10.1097/sap.0000000000000868 28792428

[24] Korolev A.I. Fedorovich A.A. Gorshkov A.Yu. Dadaeva V.A. Kim O.T. Mikhailova M.A. Vasilyev D.K. Dzhioeva O.N. Akasheva D.U. Drapkina O.M. Upper limbs skin microvascular characteristics in healthy men of working age. Profilakticheskaya meditsina 2021; 24(7):60–69, 10.17116/profmed20212407160

[25] Aksoy N. Kaplan D.S. Orkmez M. Eronat Ö. Evaluation of intestinal necrosis with laser Doppler in experimental mesenteric ischemia model. Ulus Travma Acil Cerrahi Derg 2024; 30(1):1–8, 10.14744/tjtes.2024.38399 38226574 PMC10977481

[26] Wang L. Yuan P.Q. Taché Y. Vasculature in the mouse colon and spatial relationships with the enteric nervous system, glia, and immune cells. Front Neuroanat 2023; 17: 1130169, 10.3389/fnana.2023.1130169 37332321 PMC10272736

